# Attribution of Neuropsychiatric Manifestations to Systemic Lupus Erythematosus

**DOI:** 10.3389/fmed.2018.00068

**Published:** 2018-03-14

**Authors:** Alessandra Bortoluzzi, Carlo Alberto Scirè, Marcello Govoni

**Affiliations:** ^1^Department of Medical Sciences, Section of Rheumatology, University of Ferrara, Azienda Ospedaliero-Universitaria Sant’Anna di Ferrara, Cona, Italy

**Keywords:** systemic lupus erythematosus, neuropsychiatric, attribution, neurological, psychiatric, assessment, model

## Abstract

Neuropsychiatric (NP) involvement in systemic lupus erythematosus (SLE) is one of the most severe manifestations of the disease that has a heavy impact on patient’s functioning, quality of life, and disease outcome. The prevalence is highly variable and the clinical phenotypes vary from common syndromes to rare NP entities. Its occurrence may be the result of a primary manifestation of SLE, secondary to other conditions (such as infections or metabolic disturbances) or the effect of concomitant comorbidities that often complicate the disease course. Correct attribution of NP events may pose diagnostic challenges and it is a critical factor in selecting the correct management. Although there is still no diagnostic gold standard to rightly diagnose NPSLE syndromes, great advances have been made in improving the clinician judgment in the evaluation process. In this narrative review, we present and discuss available evidence concerning NPSLE with a special focus on the attribution models developed using composite decision rules to ascribe NP events to SLE.

## Introduction

Neuropsychiatric (NP) involvement in systemic lupus erythematosus (SLE) remains one of the most challenging manifestations of the disease. Its prevalence is highly variable ranging from 14 to 75% (1). One of the major issues that makes the epidemiology of NPSLE so poorly defined is the “attribution” of NP event, a process aimed at establishing whether a physiopathologic link exists between a given NP event and the underlying disease (2). In other words, the “attribution” represents a critical step in differentiating primary NPSLE (disease-related) from secondary (disease-unrelated) NP manifestations. Intuitively this step is of outstanding importance to drive the consequential therapeutic approach. In accordance with EULAR recommendation, it is also crucial to remember that the initial diagnostic workup of each SLE patient with new or unexplained symptoms or signs suggestive of NP disease should be similar to that in non-SLE patients presenting the same manifestation(s) (3). Another important landmark was represented by the 1999 American College of Rheumatology (ACR) nomenclature which, up to now, is still the reference for the assessment of NP manifestations occurring in SLE patients (4). The ACR classification provided the definition of 19 NP syndromes, divided into central, peripheral, and autonomic ones, and defined the diagnostic criteria and the workup to ascertain each NP picture; it also listed the exclusion criteria aimed at ruling out NP event not directly related to SLE and the associated concomitant or pre-existing comorbidities to consider as potential confounding factors.

## Existing Attribution Models

Soon after the publication of the 1999 ACR nomenclature, in 2001 Ainiala et al. made a first significant step toward the attribution process performing a cross-sectional, population-based study to assess the validity of ACR nomenclature for NPSLE (5). Covering an area with 440,000 people they included and referred to a clinical neurologic examination and neuropsychological testing a total of 46 patients fulfilling the criteria for a definite diagnosis of SLE and as many controls randomly identified from the Finnish Population Register matched by age, gender, level of education, and municipality of residence. The prevalence of each manifestation listed in the ACR criteria was evaluated for SLE cases and controls. At least one NP syndrome was identified in 42 SLE patients (91%) and 25 controls (54%) with a specificity of 46% and a sensitivity of 91% among SLE patients. To improve the performance of the ACR criteria, the authors excluded anxiety, headache, mild depression, mild cognitive dysfunction (deficits in less than three of eight cognitive domains specified in the ACR case definitions) and polyneuropathy without electrophysiological confirmation deemed as highly not specific because largely represented also in the general population; with these modifications the so-called Ainiala’s revised criteria showed a specificity of 93% with a sensitivity equal to 46% in SLE cohort (5).

In 2002, systemic lupus international collaborating clinics (SLICC) set up an inception cohort to prospectively study the clinical features and outcomes of nervous system disease in patients with SLE (6, 7). In their studies, Hanly et al. adopted the ACR nomenclature for classifying NP events and applied three different simple decision rules to determine the attribution of NP events taking into account: (1) the time of onset of the NP event(s) in relation to the diagnosis of SLE; (2) the presence of concurrent “non-SLE factor” thought to be a significant contributor to the event(s) as listed in the ACR nomenclature; and (3) the occurrence of “minor” NP event(s) as defined by Ainiala et al. The time onset of the NP event identified the domain on which two different models were based: a more stringent model A with an enrollment window of 21 months including NP events observed from 6 months prior to the date of diagnosis of SLE up to 15 months following the diagnosis of SLE and a less stringent model B including NP events observed within 10 years to the date of diagnosis of SLE. The onset of NP events prior to the 6 months enrollment window for model A or the onset of NP events 10 years before the diagnosis of SLE for model B, the identification of non-SLE factors that were responsible for the NP event (“exclusion factors”), and the occurrence of a “minor” NP event as defined by Ainiala et al. were considered arguing against the attribution of a given NP event to SLE. Vice versa, an NP event falling within the time window, the absence of exclusion factors, and the occurrence of an NP event not included in the Ainiala’s list were deemed as evidence in favor of the attribution of the NP event as SLE related. By applying these rules, the proportion of NP events attributed to SLE varied between 17.9 and 30.9% (Table S1 in Supplementary Material), depending upon the more or less stringent model adopted for attribution. Regardless of attribution, in this cohort, headache, followed by mood disorders and seizures disorders were the most common NP events observed, while seizure, mood disorders, and cerebrovascular disease were the most common NP events attributed to SLE, followed by cranial neuropathy in the higher stringent model A and by cognitive dysfunctions in the lesser stringent model B (8).

In 2008, Monov and Monova introduced a simplified model for NPSLE approach which relied on the partition of two groups of criteria: a “major group” including clinical NP pictures such as seizures, psychosis, cerebrovascular event, cranial neuropathy, motor disturbances, and quantitative alterations of consciousness and a “minor group” including cognitive dysfunction, headache due to lupus, peripheral neuropathy, some instrumental information as magnetic resonance changes and electrophysiological changes, and serologic data such as anti-ribosomal-P and/or antiphospholipid autoantibodies. Excluding secondary causes, diagnosis of NPSLE could be performed in the presence of one of the major NP events from the first group or in the presence of at least two indicators from the second group of criteria. This model had a sensitivity of 90.3% and specificity of 67.7% (9).

**Figure 1:**
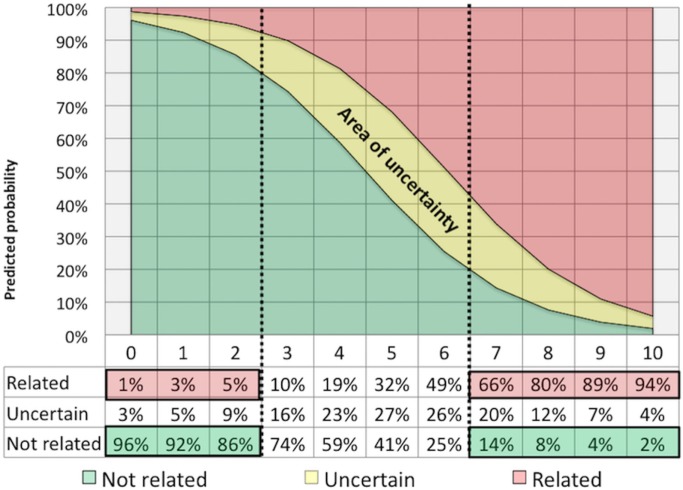
Attribution threshold: the curve synthesizes the percentage of neuropsychiatric events attributed (or not attributed) to systemic lupus erythematosus for definite cut-off values obtained with the use of the Italian algorithm applied in a combined dataset including three cohorts of NPSLE patients (13).

More recently, the Italian Study Group for NPSLE on behalf of the Italian Society of Rheumatology developed a new attribution model based on a simple numerical algorithm yielding a score ranging from 0 to 10, based on four items (see Table S2 in Supplementary Material): (1) the temporal relationship of NP events to the diagnosis of SLE (i.e., before, concomitant or after the SLE onset); (2) the presence of minor or common NP events (included in the Ainiala’s list); (3) the recognition of confounding factors (i.e., alternative etiologies or non-SLE contributing factors derived from the ACR case definitions for each of the 19 NP syndromes); and (4) the inclusion of favoring factors (i.e., clinical and non-clinical variables supporting the attribution to SLE); for this item a list of specific SLE-related risk factors as identified in the European League Against Rheumatism recommendations (3) was generated and further information considered relevant for the attribution process, identified by an expert panel, were taken into account (10, 11). In a multiphase process, the algorithm was firstly constructed on a local training cohort of 228 SLE patients and then validated on an independent external multicenter cohort including additional 221 SLE patients, with at least one NP event in their history. Headache was the most frequently observed manifestation, followed by cerebrovascular disease in both cohorts. This first step demonstrated good performance in terms of sensitivity, specificity, positive predictive value (PPV) and negative predictive value (NPV) when compared with local multidisciplinary expert clinical judgment, assumed as the “gold” reference standard (12). In a second phase, the Italian algorithm underwent a second validation process in a new external international cohort of patients with SLE from three different countries (Brazil, Canada, and Greece). The study included 243 patients with at least one NP syndrome for a total of 336 NP events. In this third cohort, mood disorder was the most frequent manifestation (16.4%), followed by headache (14.9%), and cerebrovascular disease (11.3%). Again the “clinical judgment” provided by an independent and blinded expert multidisciplinary assessor team has been used as the reference “gold standard.” The discriminating cut point based on sensitivity, specificity, PPV, and NPV were reassessed in a pooled data analysis (including all three cohorts: training, validating, and international) and in the combined dataset. Based on the ROC curve analysis, the best cut-off for discrimination (i.e., attribution threshold) was assessed both in the international validating set and in the pooled dataset. A total score ≥7 (range from 0 to 10) identified the maximum proportion of correctly classified NPSLE cases, both first and following NP events, yielding a PPV of 82.9%, NPV of 73.6% a sensitivity of 71.2%, and specificity of 84.5% (13). Overall, this model has allowed a confident correct attribution of NP events deemed as SLE related in about one-third of cases, a percentage quite similar to that found in the SLICC cohort (Figure 1). Contrary to the SLICC models, which exclude minor NP manifestations from the possibility to be attributed to SLE, the Italian Study Group model can be used to attribute both major and minor manifestations. However, due to the structure of the algorithm and to the different weights assigned to the type of event only a small percentage [less than 20% of minor manifestations (14)], reached the cut-off score to be considered as related manifestation.

In the last year, the interest for NPSLE attribution process has increased, and two new and different approaches have been suggested. A new proposal for a simplified and versatile diagnostic algorithm came from Tay and Mak (15). First, the authors proposed to eliminate NP events applying the exclusion criteria provided by the ACR nomenclature and the events listed in the Ainiala’s rules. At this point, the authors suggest to support the attribution process using the “Italian favoring factors” to finally categorize the events into three groups: (a) NPSLE manifestations concerning the central nervous system, according to the ACR nomenclature '99, (b) NPSLE manifestation concerning the peripheral nervous system, according to the ACR nomenclature '99, (c) NPSLE events excluded from the ACR nomenclature '99 such as posterior reversible encephalopathy syndrome, optical neuromyelitis, or small fibers neuropathy (16), as emerging neurological manifestation among SLE patients (17).

A few months ago Magro-Checa et al. analyzed the utility of repeated assessment in the attribution process of NP events, emphasizing the value of the multidisciplinary re-evaluation over time (18). The study was conducted on patients with NPSLE from the Leiden cohort. After a first evaluation in which each patient underwent a multidisciplinary clinical, instrumental, and laboratory assessment, the group of attending specialists made a therapeutic decision according to their own clinical judgment (Figure S1 in Supplementary Material). A re-evaluation took place 3–8 months after the first event and patients underwent the same multidisciplinary assessment by the same team as in the first visit. Based on the results of the new examinations and in the light of the acquired knowledge regarding evolution of each NP event over time (improvement/deterioration following immunosuppressive therapy, occurrence of additional NP symptoms or signs) 14.5% of patients (formerly classified as having NPSLE) have been re-evaluated as having no NPSLE and only 2.2% of NP events not originally attributed to SLE, turned out to be a manifestation of NPSLE (Figure S1 in Supplementary Material). In total, 64 events (13.8%) were reclassified after the reassessment and more NP events previously classified as NPSLE were re-assigned to the non-NPSLE group, suggesting that in clinical practice over-diagnosis of NPSLE and related immunosuppressive strategy is more common than under diagnosis. This observation strengthened the need for a correct attribution “*ab initio*” to limit the risk of inappropriate exposure to immunosuppressive therapy and to related side effects.

## External Independent Validation

The importance of the issue of attribution is attested by two experiences of independent validation of the existing attribution models previously described. In 2015, Fanouriakis et al. retrospectively tested the performance of the SLICC A and B models and the Italian attribution algorithm against clinical judgment in a total of 242 NP manifestations experienced by 191 patients, from two national tertiary referral centers (Heraklion, Greece and Cluj, Romania) (14). According to physician judgment, 136 manifestations were attributed to SLE. Applying the SLICC models, 35/242 (14.5%) of events were attributed to SLE with model A and 69/242 (28.5%) with model B. Compared with physician judgment, both models showed high specificity but poor sensitivity (Table 1), suggesting that only a minority of NP manifestations considered as SLE related by treating physicians would be captured by the SLICC models. Analyzing the individual components of the SLICC models, the authors observed that the sensitivity increased much more excluding the item “time.” SLICC model A compared with model B, showed a better performance, with a specificity >70%, for CVD, cognitive dysfunction, seizures disorder, mood disorder, psychosis, cranial neuropathy, while it was not possible to test it in minor manifestations (headache and anxiety). The authors tested also the Italian attribution algorithm and the values ≥7 showed the best combination of sensitivity and specificity. Using this cut-off value, 82.4% of manifestations related to SLE according to clinical judgment had a score of ≥7, when compared with 17.0% of manifestations unrelated to SLE. Observing individual NP manifestation, the Italian algorithm showed a lower specificity only for CVD (57.1%), while for all the other manifestation the specificity ranged from 72.4% (cognitive dysfunctions) to 100% (psychosis, cranial neuropathy, headache, and anxiety disorder). Magro-Checa et al. conducted a similar prospective independent validation in patient with SLE and NP events from the Leiden cohort (18). 56 out of 463 events and 139 out of 463 were attributed to SLE with the SLICC model A and B versus176/463 events with the Italian algorithm (Table 1).

**Table 1:** Sensitivity and specificity of attribution models tested in two independent cohorts at the time of first neuropsychiatric event.

Independent cohort		Sensitivity (%)	Specificity (%)
Leiden prospective NPSLE cohort (18)	SLICC model A	33	98
	SLICC model B	64	86
	Italian algorithm (score ≥7)	83	84

Heraklion and Cluj retrospective NPSLE cohort (14)	SLICC model A	23	96
	SLICC model B	35	79
	Italian algorithm (score ≥7)	82	83

These independent experiences suggest that attribution models can be useful to support NPSLE diagnosis in routine clinical practice, especially for physicians and centers with less experience in the difficult field of NPSLE (19). Nevertheless, there are some limitations for both instruments to consider. SLICC models have not been tested against “clinical judgment” and to guarantee a good specificity it did not consider minor events that are usually more common and difficult to attribute. Italian algorithm is less satisfying in minor and diffuse events and some rare NP events are under-represented or not represented at all across different cohorts, so in these events, the reproducibility of the algorithm cannot be demonstrated.

## Key Messages, Next Steps, and Future Perspective

The development of different models offers the opportunity to integrate ACR case definition rules supporting—without replacing—clinicians in the attribution decision. Despite its inherent subjectivity, in fact the “physician judgment” continues to be the gold standard for the diagnosis of NPSLE. The added value of the new models lies in the opportunity to classify NPSLE more objectively and perhaps in the future, they could be useful tools for patients’ selection in RCT. The major limit of the discussed algorithm is their sub-optimal performance in the attribution of common minor NP events. Nevertheless, as these syndromes heavily influence the prevalence of NPSLE itself, their complete elimination from the related NPSLE pictures could seem an oversimplification of the problem. In our opinion, the inclusion of these minor manifestations in the definition of NPSLE should be retained, provided a careful and rigorous clinical evaluation and even more stringent attribution rules. In the near future, it is conceivable that research efforts in the field of advanced neuroimaging techniques and novel studies on serological and cerebrospinal fluid biomarkers will increase the sensitivity and specificity of the available attribution models, further improving their performance.

Finally, it is clear that to integrate the rules of each algorithm, considering exclusion and inclusion criteria, is a very time-consuming activity. With the final aim to have a rapid integration of mobile devices into clinical practice, we have developed a medical software application, or “app” dedicated to the Italian algorithm. The mobile app “neurolupus” is a software program, for medical use only, that runs on smartphones and other mobile devices dedicated to health care professional involved in NPSLE. The mobile app is accessible for free after registration available at this link *https://neurolupus.ospfe.it*. The app does not involve the use of sensitive data and makes the attribution process friendlier (see in Figure S2 in Supplementary Material the homepage and the registration form of the app “neurolupus”).

In conclusion, correct attribution of NPSLE syndromes may pose diagnostic challenges and it is a critical factor in selecting the correct management. Beside these considerations and although there is still no diagnostic gold standard for NPSLE, great advances have been made in improving the clinician judgment in the diagnostic process. The future perspective will be to verify whether more stringent cut points could also serve as decisional “therapeutic threshold” to position the proper strategy (to treat or not to treat) for each NPSLE syndrome.

## Author Contributions

AB was responsible for the literature review and paper writing. CS and MG were responsible for supervision and critical review of the final document for submission. All authors contributed to manuscript revision, read, and approved the submitted version.

## Conflict of Interest Statement

The authors declare that the research was conducted in the absence of any commercial or financial relationships that could be construed as a potential conflict of interest.

## References

[1] Unterman A Nolte JES Boaz M Abady M Shoenfeld Y Zandman-Goddard G. Neuropsychiatric syndromes in systemic lupus erythematosus: a meta-analysis. Semin Arthritis Rheum (2011) 41:1–11. 10.1016/j.semarthrit.2010.08.001 20965549

[2] Govoni M Bortoluzzi A Padovan M Silvagni E Borrelli M Donelli F The diagnosis and clinical management of the neuropsychiatric manifestations of lupus. J Autoimmun (2016) 74:41–72. 10.1016/j.jaut.2016.06.013 27427403

[3] Bertsias GK Ioannidis JPA Aringer M Bollen E Bombardieri S Bruce IN EULAR recommendations for the management of systemic lupus erythematosus with neuropsychiatric manifestations: report of a task force of the EULAR standing committee for clinical affairs. Ann Rheum Dis (2010) 69:2074–82. 10.1136/ard.2010.130476 20724309

[4] The American College of Rheumatology nomenclature and case definitions for neuropsychiatric lupus syndromes. Arthritis Rheum (1999) 42:599–608. 10.1002/1529-0131(199904) 10211873

[5] Ainiala H Hietaharju A Loukkola J Peltola J Korpela M Metsänoja R Validity of the new American College of Rheumatology criteria for neuropsychiatric lupus syndromes: a population-based evaluation. Arthritis Rheum (2001) 45:419–23. 10.1002/1529-0131(200110)45:5<419::AID-ART360>3.0.CO;2-X 11642640

[6] Hanly JG Urowitz MB Sanchez-Guerrero J Bae SC Gordon C Wallace DJ Neuropsychiatric events at the time of diagnosis of systemic lupus erythematosus: an international inception cohort study. Arthritis Rheum (2007) 56:265–73. 10.1002/art.22305 17195230

[7] Hanly JG The neuropsychiatric SLE SLICC inception cohort study. Lupus (2008) 17:1059–63. 10.1177/0961203308097568 19029272

[8] Hanly JG Urowitz MB Su L Gordon C Bae S-C Sanchez-Guerrero J Seizure disorders in systemic lupus erythematosus. Ann Rheum Dis (2012) 71:1502–9. 10.1136/annrheumdis-2011-201089 22492779 PMC4656036

[9] Monov S Monova D. Classification criteria for neuropsychiatric systemic lupus erythematosus: do they need a discussion? Hippokratia (2008) 12:103–7. 18923663 PMC2464312

[10] Padovan M Castellino G Bortoluzzi A Caniatti L Trotta F Govoni M Factors and comorbidities associated with central nervous system involvement in systemic lupus erythematosus: a retrospective cross-sectional case–control study from a single center. Rheumatol Int (2010) 32:129–35. 10.1007/s00296-010-1565-4 20676648

[11] Govoni M Bombardieri S Bortoluzzi A Caniatti L Casu C Conti F Factors and comorbidities associated with first neuropsychiatric event in systemic lupus erythematosus: does a risk profile exist? A large multicentre retrospective cross-sectional study on 959 Italian patients. Rheumatology (2012) 51:157–68. 10.1093/rheumatology/ker310 22075066

[12] Bortoluzzi A Scirè CA Bombardieri S Caniatti L Conti F De Vita S Development and validation of a new algorithm for attribution of neuropsychiatric events in systemic lupus erythematosus. Rheumatology (2015) 54:891–8. 10.1093/rheumatology/keu384 25339643

[13] Bortoluzzi A Fanouriakis A Appenzeller S Costallat L Scirè CA Murphy E Validity of the Italian algorithm for the attribution of neuropsychiatric events in systemic lupus erythematosus: a retrospective multicentre international diagnostic cohort study. BMJ Open (2017) 7:e015546. 10.1136/bmjopen-2016-015546 28554934 PMC5730002

[14] Fanouriakis A Pamfil C Rednic S Sidiropoulos P Bertsias G Boumpas DT. Is it primary neuropsychiatric systemic lupus erythematosus? Performance of existing attribution models using physician judgment as the gold standard. Clin Exp Rheumatol (2016) 34:910–7. 27463840

[15] Tay SH Mak A. Diagnosing and attributing neuropsychiatric events to systemic lupus erythematosus: time to untie the Gordian knot? Rheumatology (2017) 56:i14–23. 10.1093/rheumatology/kew338 27744358

[16] Bortoluzzi A Scirè CA Govoni M Comment on: diagnosing and attributing neuropsychiatric events to systemic lupus erythematosus: time to untie the Gordian knot? Rheumatology (2017) 56:856–7. 10.1093/rheumatology/kex017 28339930

[17] Barber CE Leclerc R Gladman DD Urowitz MB Fortin PR. Posterior reversible encephalopathy syndrome: an emerging disease manifestation in systemic lupus erythematosus. Semin Arthritis Rheum (2011) 41:353–63. 10.1016/j.semarthrit.2011.07.001 21868061

[18] Magro-Checa C Zirkzee EJ Beaart-van de Voorde LJJ Middelkoop HA van der Wee NJ Huisman MV Value of multidisciplinary reassessment in attribution of neuropsychiatric events to systemic lupus erythematosus: prospective data from the Leiden NPSLE cohort. Rheumatology (2017) 56:1676–83. 10.1093/rheumatology/kex019 28339952

[19] Govoni M Bortoluzzi A Lupus or not lupus? Neuropsychiatric symptom attribution in systemic lupus erythematosus. Rheumatology (2017) 56(10):1639–40. 10.1093/rheumatology/kex178 28541514

