# Supplementary material for: Attribution of Neuropsychiatric Manifestations to Systemic Lupus Erythematosus
Source: Front Med (Lausanne). 2018 Mar 14;5:68. doi: 10.3389/fmed.2018.00068 (PMC5861139; doi:10.3389/fmed.2018.00068)

Supplementary Material

Attribution of neuropsychiatric manifestations to SLE

Alessandra Bortoluzzi[1*], Carlo Alberto Scirè[1], Marcello Govoni[1]

[1]Department of Medical Sciences, Section of Rheumatology, University of Ferrara and Azienda Ospedaliero-Universitaria Sant'Anna di Ferrara, Via Aldo Moro 8, 44124 Cona, Italy.

***Correspondence:**

Corresponding Author
brtlsn1@unife.it

.

1 Supplementary Figures and Tables

1.1 Supplementary Figures Legends

Supplementary Figure 1. The multidisciplinary approach to NPSLE proposed by Magro-Checa et al. In a patient with a suspected NPSLE a multidisciplinary team re-evaluated each manifestation after a course of treatment assigning the proper re-attribution according to therapeutic response (modified from Magro-Checa et al. (18)).

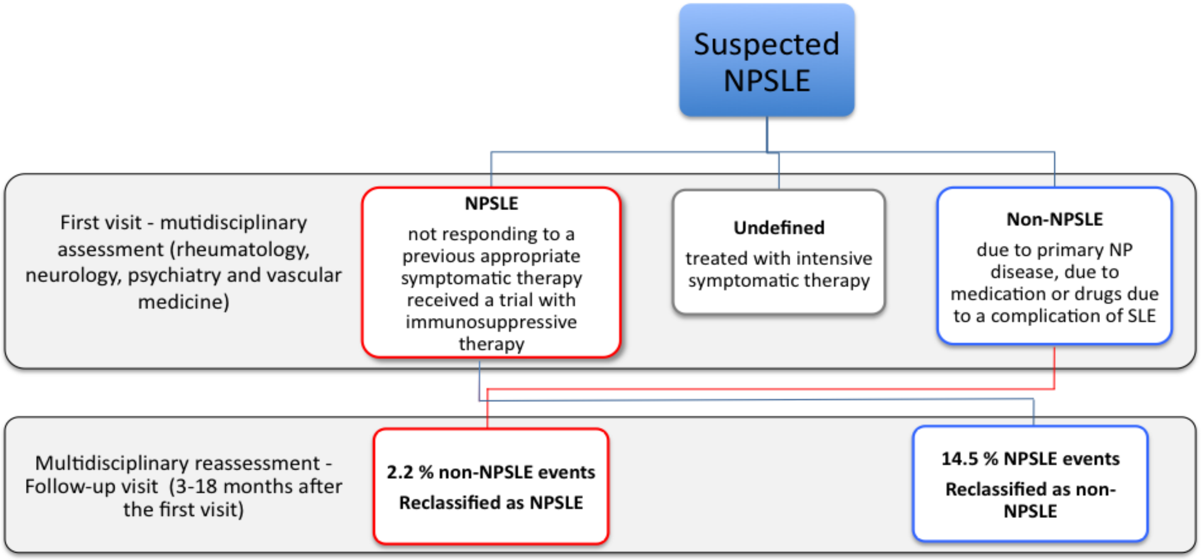

Supplement: Supplementary file 1 [file Image_1.PDF]
