# Supplementary material for: Attribution of Neuropsychiatric Manifestations to Systemic Lupus Erythematosus
Source: Front Med (Lausanne). 2018 Mar 14;5:68. doi: 10.3389/fmed.2018.00068 (PMC5861139; doi:10.3389/fmed.2018.00068)
Supplement: Supplementary file 3 [file table_1.docx]

Supplementary Material

Attribution of neuropsychiatric manifestations to SLE

Alessandra Bortoluzzi^1*^, Carlo Alberto Scirè^1^, Marcello Govoni^1^

^1^Department of Medical Sciences, Section of Rheumatology, University of Ferrara and

Azienda Ospedaliero-Universitaria Sant’Anna di Ferrara, Via Aldo Moro 8, 44124 Cona, Italy.

***Correspondence:**

Corresponding Author
brtlsn1@unife.it

.

**
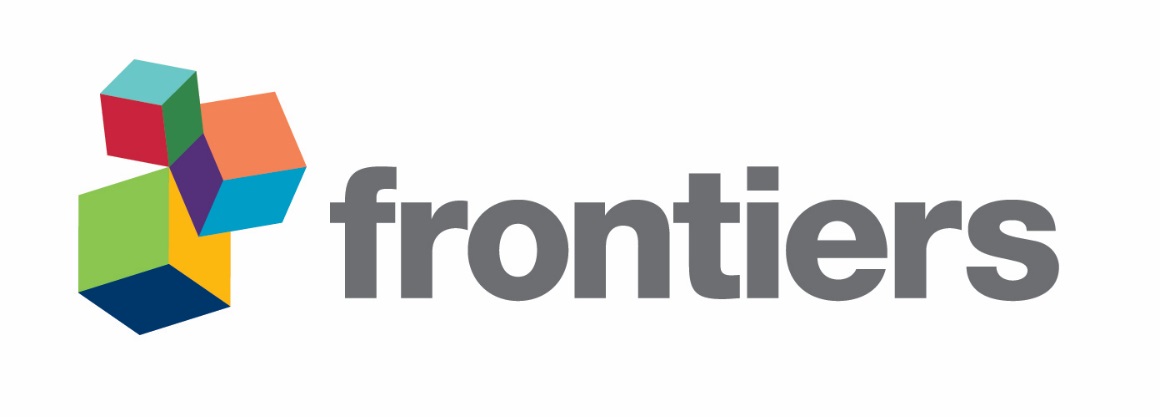
**

## Supplementary Tables

**Supplementary Table 1.** Summary of the SLICC inception cohort experience. The models A (more stringent) and B (less stringent) allowed a confident proper attribution of NP events deemed as SLE related ranging from 17 to 31 % of the cases.

|  | No. pts | No. of pts. with at least 1 NP event | No. NP events | Percentage of NP events attributed to SLE with model A and B (%) |
| --- | --- | --- | --- | --- |
| Hanly et al, 2008 (1) | 420 | 133 | 214 | 15 - 36 |
| Hanly et al, 2007 (2) | 572 | 158 | 242 | 19 - 38 |
| Hanly et al, 2008 (3) | 890 | 271 | 407 | 16.5 - 33.9 |
| Hanly et al, 2010 (4) | 1206 | 486 | 843 | 17-30 |
| Hanly et al, 2012 (5) | 1631 | 747 | 1358 | 16.9 - 29.9 |
| Hanly et al, 2013 (6) | 1732 | 788 | 1455 | 16.8 - 30.5 |
| Hanly et al, 2015 (7) | 1827 | 863 | 1627 | **17.8 - 30.9** |

References

1. Hanly JG, Urowitz MB, Siannis F, Farewell V, Gordon C, Bae SC, Isenberg D, Dooley MA, Clarke A, Bernatsky S, et al. Autoantibodies and neuropsychiatric events at the time of systemic lupus erythematosus diagnosis: results from an international inception cohort study. *Arthritis Rheum* (2008) **58**:843–853. doi:10.1002/art.23218

2. Hanly JG, Urowitz MB, Sanchez-Guerrero J, Bae SC, Gordon C, Wallace DJ, Isenberg D, Alarcón GS, Clarke A, Bernatsky S, et al. Neuropsychiatric events at the time of diagnosis of systemic lupus erythematosus: an international inception cohort study. *Arthritis Rheum* (2007) **56**:265–273. doi:10.1002/art.22305

3. Hanly JG, Urowitz MB, Su L, Sanchez-Guerrero J, Bae SC, Gordon C, Wallace DJ, Isenberg D, Alarcón GS, Merrill JT, et al. Short-term outcome of neuropsychiatric events in systemic lupus erythematosus upon enrollment into an international inception cohort study. *Arthritis Rheum* (2008) **59**:721–729. doi:10.1002/art.23566

4. Hanly JG, Urowitz MB, Su L, Bae SC, Gordon C, Wallace DJ, Clarke A, Bernatsky S, Isenberg D, Rahman A, et al. Prospective Analysis Of Neuropsychiatric Events In An International Disease Inception Cohort of SLE Patients. *Ann Rheum Dis* (2010) **69**:529–535. doi:10.1136/ard.2008.106351

5. Hanly JG, Urowitz MB, Su L, Gordon C, Bae S-C, Sanchez-Guerrero J, Romero-Diaz J, Wallace DJ, Clarke AE, Ginzler EM, et al. Seizure disorders in Systemic Lupus Erythematosus. *Ann Rheum Dis* (2012) **71**:1502–1509. doi:10.1136/annrheumdis-2011-201089

6. Hanly JG, Urowitz MB, O’Keeffe AG, Gordon C, Bae S-C, Sanchez-Guerrero J, Romero-Diaz J, Clarke AE, Bernatsky S, Wallace DJ, et al. Headache in systemic lupus erythematosus: results from a prospective, international inception cohort study. *Arthritis Rheum* (2013) **65**:2887–2897. doi:10.1002/art.38106

8. Hanly JG, Su L, Urowitz MB, Romero-Diaz J, Gordon C, Bae S-C, Bernatsky S, Clarke AE, Wallace DJ, Merrill JT, et al. Mood Disorders in Systemic Lupus Erythematosus: Results From an International Inception Cohort Study. *Arthritis Rheumatol Hoboken NJ* (2015) **67**:1837–1847. d
