# Supplementary material for: Attribution of Neuropsychiatric Manifestations to Systemic Lupus Erythematosus
Source: Front Med (Lausanne). 2018 Mar 14;5:68. doi: 10.3389/fmed.2018.00068 (PMC5861139; doi:10.3389/fmed.2018.00068)
Supplement: Supplementary file 4 [file table_2.docx]

Supplementary Material

Attribution of neuropsychiatric manifestations to SLE

Alessandra Bortoluzzi^1*^, Carlo Alberto Scirè^1^, Marcello Govoni^1^

^1^Department of Medical Sciences, Section of Rheumatology, University of Ferrara and

Azienda Ospedaliero-Universitaria Sant’Anna di Ferrara, Via Aldo Moro 8, 44124 Cona, Italy.

***Correspondence:**

Corresponding Author
brtlsn1@unife.it

.

**
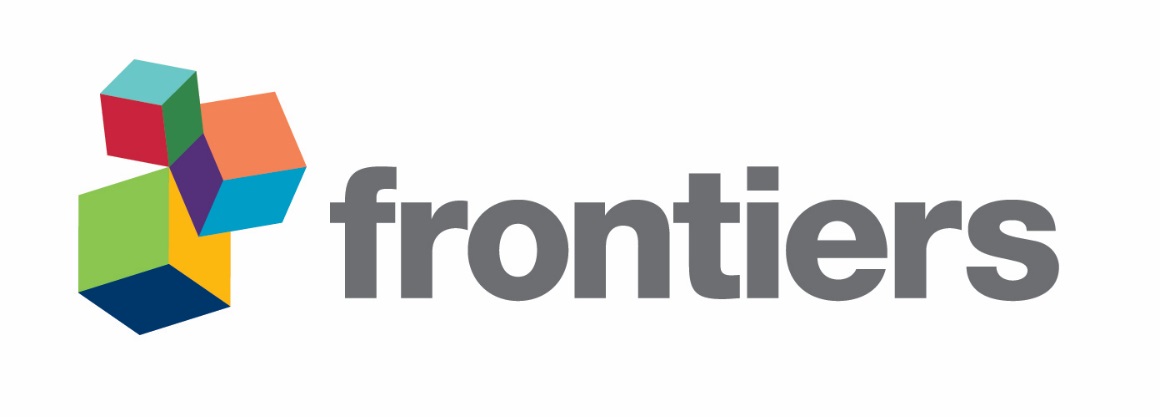
**

**Supplementary Table 2.** Italian attribution model.

| **Item** | Score |
| --- | --- |
| **1. Time of the onset of NP event with respect to SLE clinical onset**  Before (>6 months before SLE onset) 0  Concomitant (within 6 months of SLE onset) 3  After (>6 months after SLE onset) | 0  3  2 |
| **2. Minor or not specific NP events as defined by Ainiala et al. (5)**  Present (i.e. minor or common NP events as proposed by Ainiala et al.)  Absent (i.e. NP events other than those proposed by Ainiala et al.) | 0  3 |
| **3. Confounding factors or not SLE-related associations as defined by the ACR glossary**  None or not applicable  Present (one confounding factor)  Present (more than one confounding factor) | 2  1  0 |
| **4. Favouring factors**  None or not applicable  Present (one additional or favouring factor)  Present (more than one additional or favouring factor) | 0  1  2 |
